# Self-Supported Crack-Free Conducting Polymer Films with Stabilized Wrinkling Patterns and Their Applications

**DOI:** 10.1038/srep36686

**Published:** 2016-11-09

**Authors:** Jixun Xie, Xue Han, Haipeng Ji, Juanjuan Wang, Jingxin Zhao, Conghua Lu

**Affiliations:** 1 School of Materials Science and Engineering, Tianjin University, Tianjin 300072, P. R. China

## Abstract

Self-supported conducting polymer films with controlled microarchitectures are highly attractive from fundamental and applied points of view. Here a versatile strategy is demonstrated to fabricate thin free-standing crack-free polyaniline (PANI)-based films with stable wrinkling patterns. It is based on oxidization polymerization of pyrrole inside a pre-wrinkled PANI film, in which the wrinkled PANI film is used both as a template and oxidizing agent for the first time. The subsequently grown polypyrrole (PPy) and the formation of interpenetrated PANI/PPy networks play a decisive role in enhancing the film integrity and the stability of wrinkles. This enhancing effect is attributed to the modification of internal stresses by the interpenetrated PANI/PPy microstructures. Consequently, a crack-free film with stable controlled wrinkles such as the wavelength, orientation and spatial location has been achieved. Moreover, the wrinkling PANI/PPy film can be removed from the initially deposited substrate to become free-standing. It can be further transferred onto target substrates to fabricate hierarchical patterns and functional devices such as flexible electrodes, gas sensors, and surface-enhanced Raman scattering substrates. This simple universal enhancing strategy has been extended to fabrication of other PANI-based composite systems with crack-free film integrity and stabilized surface patterns, irrespective of pattern types and film geometries.

The ability to pattern polymer surfaces at different length scales is highly important in a wide variety of applications ranging from microelectronics^1^ to biotechnology^2^. Several strategies have been developed to fabricate structured surfaces, which can be roughly categorized into two groups: top-down and bottom-up^3^. Till now, various functions of patterned polymer surfaces have been obtained through these techniques, such as lithography-based techniques and self-assembly routes^4^,^5^,^6^. Recently, thin free-standing functional polymer films with well-defined micro/nanostructures have attracted increasing attention, because they could be arbitrarily folded into different shapes and simply transferred to desirable target substrates for the prescribed applications such as optical detection, micro-actuator, and controlled release^7^. However, how to realize the large-area preparation of patterned free-standing films with a good film integrity is still a great challenge.

As a typically non-lithographic micro/nano-fabrication method, surface wrinkling has attracted intense interest owing to its simplicity, universality, low cost and so on^8^,^9^,^10^,^11^. This instability-driven patterning usually occurs on a film/substrate bilayer system with a rigid film bound to a compliant substrate (e.g., polydimethylsiloxane (PDMS)), once the driving force exceeds the bilayer material-defined critical value. The driving force for surface wrinkling comes from the strain mismatch between the film and the substrate due to their differences in Young’s moduli, thermal expansion coefficients or solvent swelling coefficients, when the bilayer system is subjected to mechanical stretching/compressing, heating/cooling, or solvent swelling/de-swelling, respectively^9^,^12^. It should be pointed out that surface wrinkling morphologies (e.g., wavelength, amplitude and orientation) are sensitive to the localized stress field^9^,^12^. Based on this intrinsic stress-relief characteristic, different surface wrinkling morphologies (e.g. herringbone, labyrinth and dimple) and switchable wrinkled/dewrinkled surfaces have been obtained by changing the stress state of the film/substrate system^13^,^14^,^15^. However, these instable patterns have internal limitations when involved in specific fields such as friction^16^ and contact^17^. This stress-relief feature of the wrinkling patterns could be an obvious disadvantage in the case where surface patterns should be maintained. Furthermore, owing to the instability-driven mechanism, the surface wrinkling patterns strongly depend on the constraint of the underlying substrate and they may disappear when the substrate is removed. However, little attention has been paid to enhancing the “stability” of wrinkling patterns and to fabricating substrate-free self-supported wrinkling films.

On the other hand, conducting polymers (CPs) hold a variety of advantages such as good electrical conductivity, excellent optoelectronic properties and low energy optical transitions^18^,^19^,^20^. Due to these attractive properties, CPs have been applied in a number of fields including flexible electronics^21^,^22^, sensors^23^,^24^, photodetectors^25^ and energy storage^21^,^26^,^27^. Interestingly, it has been identified that surface patterns could greatly enhance the performances (e.g., gas sensing properties) and extend the application fields of CPs^28^,^29^. For example, patterned CPs films have been used as promising platforms for study of cell attachment and growth, tissue regeneration, and so on^30^,^31^. Furthermore, novel properties of CPs-based hybrid materials can be obtained due to their coupling effects^32^,^33^. Among these CPs, polyaniline (PANI) and polypyrrole (PPy) have been extensively investigated because of their simple synthesis and excellent environmental stability^34^. In particular, PANI has intriguing doping states and redox levels (e.g., protonated emeraldine salt (ES), leucoemeraldine base (LEB), pernigraniline base (PB), emeraldine base (EB))^20^,^34^. These doping states and redox levels with distinct chemical reactivity can be mutually converted under suitable conditions, which is of great importance to fabricate smart PANI-based composite systems^15^,^35^.

In this study, we demonstrate a novel facile method to fabricate patterned self-supported crack-free conducting films at a large-area scale. Here a pre-wrinkled PANI film on a PDMS substrate is used both as a template and oxidizing agent to direct the oxidative polymerization of pyrrole for the first time. The wrinkling patterns is reinforced by the additional growth of PPy inside the as-wrinkled PANI film and the formation of interpenetrated PANI/PPy networks, which modifies the stresses of the wrinkling system and stabilizes the wrinkling morphologies and simultaneously inhibits the crack formation. Owing to the reinforcing effect, the wrinkling patterns on the conducting film is maintained steadily even when the film is peeled from the PDMS substrate. Thus, a patterned, free-standing conducting film with the crack-free film integrity is obtained at the large-area scale. We further take advantage of the reductive ability of PANI in the PANI/PPy composite wrinkling film to prepare closely-packed metal nanoparticles (e.g., Au and Ag). We demonstrate that the free-standing patterned PANI-based composite films, which can be transferred onto target substrates, have great promises in the fields of flexible electrodes, gas sensors and surface-enhanced Raman scattering substrates. Therefore, this work will pave the way to fabricate various self-supported crack-free patterned films for multi-functions and unprecedented applications.

## Results

**Figure 1:**
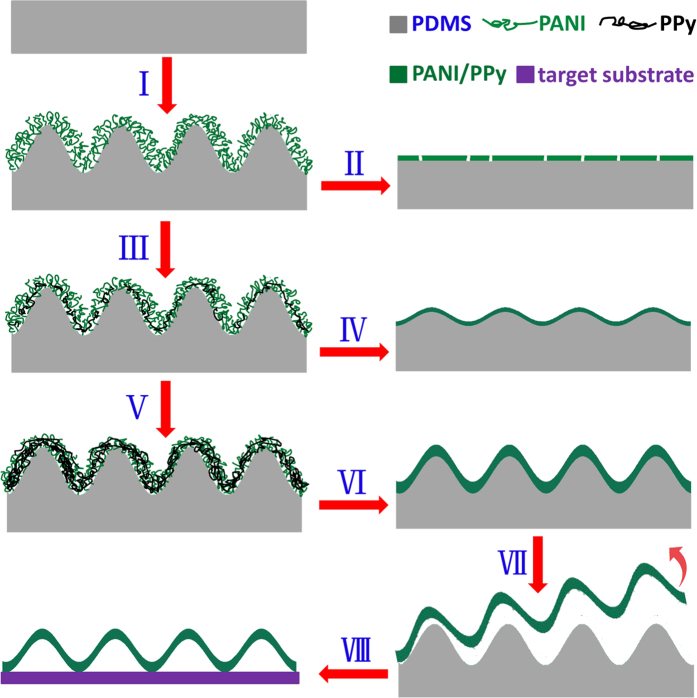
Schematic illustration of the process for the fabrication of a free-standing wrinkled PANI-based film and the effect of the additional deposition of PPy on the pattern stability and film integrity: *in-situ* self-wrinkling PANI film which is grown on a PDMS_(n:1)_ substrate (I), followed by air drying (II); the additional growth of PPy inside the wrinkled PANI film to fabricate a PDMS_(n:1)_/PANI/PPy film with a relatively low (III) and high (V) content of PPy, followed by air drying (IV and VI), as well as peeling from the PDMS_(n:1)_ substrate to obtain a free-standing one (VII) and further transferring it onto target substrates (VIII).

As illustrated in Fig. 1, a free-standing PANI-based film with wrinkling patterns is fabricated through the following processes. First, *in-situ* self-wrinkling of PANI film occurs during oxidizing polymerization of aniline on a PDMS_(n:1)_ substrate (I). Generally, the disappearance of surface wrinkles and appearance of cracks happen once the wrinkled PDMS_(n:1)_/PANI film is dried (II). Subsequently, the wrinkled PANI film is converted into the fully oxidized state of pernigraniline base (PB), and then acts both as the template and oxidizing agent to induce the oxidative polymerization of pyrrole into PPy (III). As a result, the PDMS_(n:1)_/PANI/PPy film composed of the interpenetrated PANI/PPy network is generated and the stability of the wrinkle patterns as well as the film integrity is greatly improved. The enhancing effect is strongly dependent on the additionally deposited content of PPy (III–VI). The stabilized PDMS_(n:1)_/PANI/PPy film can be peeled from the PDMS_(n:1)_ substrate to obtain a free-standing crack-free film with well reserved microstructures (VII), which can be further transferred to target surfaces/substrates (VIII). Here unless specified otherwise, the applied PDMS_(n:1)_ substrate is from the weight ratio (n:1) of base/curing agent of 10:1 (i.e., n:1 = 10:1).

### Formation of a wrinkled crack-free PANI/PPy film

**Figure 2:**
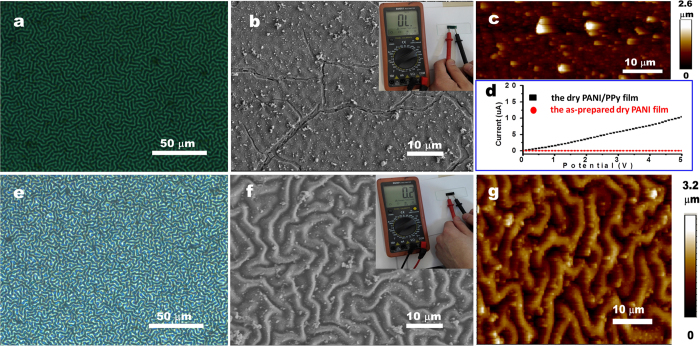
Optical (**a,e**), SEM (**b,f**) and AFM (**c,g**) images of the as-prepared PDMS/PANI film (**a–c**) and the PDMS/PANI/PPy film (**e–g**). The film: in the wet state (**a,e**) and in the dry state (**b–d,f,g**).

The self-wrinkling PANI film is fabricated by *in-situ* growth of PANI on the PDMS substrate via oxidative polymerization of aniline in the mixed HCl/aniline/ ammonium persulfate (APS) solution, which has been investigated in our previous reports^15^,^36^. As shown in Fig. 2a, after the polymerization at 2~5 °C for 12 min, labyrinth wrinkles with the average wavelength of 3.1 ± 0.1 μm are induced on the wet green PANI film. The green PANI film is in the ES state, supported by the film color and the subsequently recorded UV-vis absorption spectrum with the domain absorption peaks at about 360 nm and 820 nm^36^,^37^. A swelling-induced self-wrinkling mechanism has been identified to be responsible for the formation of wrinkling patterns on the grown PANI film^36^. Roughly speaking, different swelling capability in the acid medium between the grown PANI film and the underlying PDMS substrate results in the *in-situ* self-wrinkling^36^. When the as-prepared PDMS/PANI film is subjected to the air-drying processing (Fig. S1), cracks appear increasingly. Simultaneously, the original surface wrinkling patterns become unclear. Consequently, the wrinkle morphology is nearly invisible in the dried PDMS/PANI film from the recorded SEM image (Fig. 2b) and AFM image (Fig. 2c). In our experiments, except for the samples used for the *in-situ* observation, other samples were subjected to the air-drying processing at room temperature (about 25 °C) for 6 h with the relative humidity (RH) of 35%. Because of the presence of a large number of surface cracks, the conductivity of the dry PDMS/PANI film is very poor (inset of Fig. 2b,d), which will restrict its applications seriously. Indeed, the crack-free PANI films in the dry state are widely needed in various applications, such as anti-corrosion coatings, sensors, and super-hydrophobic surfaces. Especially if the wrinkling patterns are stable in the dry PANI film, they can be free-standing and further transferred to target substrates for the functional device fabrication. To avoid the occurrence of these devastating cracks, it is necessary to find out how these cracks take place. In our system, the wet grown PANI film on the PDMS is in the swelling state when immersed in the acid solution. In this case, no crack is observed because of the swollen PANI film with a small modulus and the relatively low stress applied on the wet film. However, when taken out of the solution with water removal from the ES state of the swollen PANI film, the PANI film undergoes the swelling to de-swelling transformation, accompanied with the film contraction. The above transformation brings the strain on the PANI film. Meanwhile, the water evaporation leads the wet PANI film to the dried one. Due to the intrinsic brittle and rigid nature of the dried PANI film, its fracture strain/stress is very low. When the above contraction-induced strain/stress exceeds the fracture ones, cracks will appear. From Fig. S1, we can see that the film’s crack opening becomes wider during water removal from the film. It is because that more water evaporation makes the film more contraction, and thus leads to the increasing applied strain/stress. As the water evaporates, the original swelling PANI film becomes de-swelling, along with the decrease of the compressive stress/strain that drives to form wrinkles. As a result, the wrinkle amplitudes reduce gradually (e.g., wrinkles become unclear under the optical observation, Fig. S1). The as-formed cracks would promote the further release of the internal stress and the decrease in the wrinkle amplitude. Interestingly, the cracked film seems to be “self-healing” when it is exposed to 1 M HCl solution (Fig. S2). From the recorded *in-situ* observations, we see the crack widths decrease gradually. Finally, cracks seem to vanish companied by the re-occurrence of surface wrinkling in the PANI film. Evidently, the dewrinkled PANI film in the dry state is again swollen in the HCl solution. The cracked parts contact each other to form an “integrated” film (Fig. S2d). Note that the swelling-induced surface wrinkling and the deswelling-induced de-wrinkling can be also cycled.

The above *in-situ* optical observations during water evaporation and HCl diffusion (Figs S1 and S2) indicate that the wet swollen PANI film on the PDMS substrate can accommodate some substances (e.g., solvent and monomers). In return, the incorporated monomers will modify the internal stress of the PDMS/PANI wrinkling system, and thus stable wrinkles coupled with the crack-free film will be available, once the embedded monomers are polymerized^17^. Here we make good use of the fully oxidized PB state of PANI to direct the oxidative polymerization of pyrrole inside the above PANI film, considering the relationship of the redox potential between PB (0.8 V)^35^ and PPy(~0.5 V)^38^. Indeed, it has been reported that the fully oxidized PB state of PANI can be used as an oxidizing agent to induce the oxidative polymerization of some monomers (e.g., aniline)^35^. For simplicity, the resulting sample is named as the PDMS/PANI/PPy film. Just as expected, the additionally deposited PPy has a remarkable effect on the film integrity and the wrinkling patterns (Fig. 2e,f). Compared with the dried PDMS/PANI film with a large number of cracks and de-wrinkled morphologies (Figs S1 and S3a,b), the air-dried PDMS/PANI/PPy film is free of cracks as well the wrinkle patterns have been well reserved. Furthermore, even when the PDMS/PANI/PPy sample was treated with vacuum drying at 100 °C for 12 h, there was no crack in the wrinkled PANI/PPy film yet, which further shows the good stability of the wrinkled patterns we have fabricated. Owing to the good film integrity, the dried PDMS/PANI/PPy film has excellent electrical conductivity (Fig. 2d and inset of Fig. 2f), which is in sharp contrast with the dried PDMS/PANI film (Fig. 2c). As for the wrinkle patterns retained on the PDMS/PANI/PPy film (Fig. 2e–g), the average wavelength is basically equal to the *in-situ* self-wrinkling PDMS/PANI film shown in Fig. 2a.

### Examination of experimental conditions to reveal the enhancing mechanism for the stabilized wrinkles

**Figure 3:**
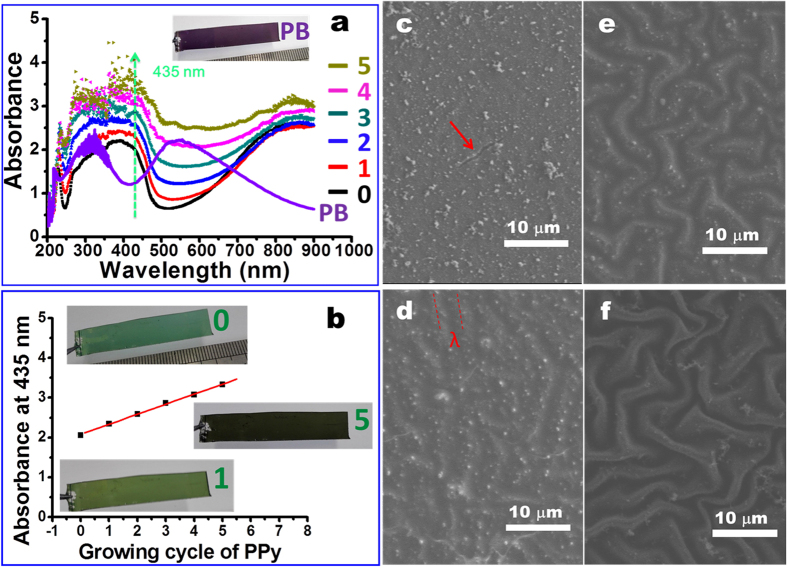
Effects of the growing cycle of the additional PPy deposition on the UV-vis absorbance spectrum (**a**), the absorbance at 435 nm (**b**), film color (inset in a,b), and SEM images (**c–f**) of the resulting PDMS_(40:1)/_PANI/PPy film. Cycle number: 0 (**c**); 2 (**d**); 4 (**e**); 10 (**f**). For comparison, the UV-vis absorption spectrum of the PDMS/PANI film with the PB state of the PANI film was demonstrated in frame a.

In order to reveal the underlying physics responsible for the notable enhancement both in the film integrity and the wrinkle stability by the additional PPy growth, we investigate the influence of the deposited PPy amount. Firstly, we study how to control the PPy content grown in the PANI film. Here the PB state of the PANI film is obtained by exposure of the ES state of the self-wrinkling PANI film to the strong oxidative APS solution for 3 s. The film color changes from the initial green into the final purple (inset of Fig. 3a). Correspondingly, the domain absorption peaks (i.e., 360 nm and 820 nm) of the ES state^38^ vanish, and two new absorption peaks at 334 nm (π−π* transition) and 550 nm (exciton transition) assigned to the PB state of PANI^39^,^40^ appear instead (Fig. 3a and inset of Fig. 3b). Simultaneously, the wrinkling surface is converted into the de-wrinkling surface (Fig. S3a,b). When the PB state of PANI film is immersed in the mixed solution composed of 0.1 M pyrrole and 1 M HCl without any other oxidizing agent added, oxidative polymerization of pyrrole into PPy is triggered. As a result, the absorbance at ~435 nm assigned to the main absorption peak of PPy^41^,^42^ is enhanced, compared to the initial PDMS/PANI film (Fig. 3a).

We think three reactions are involved when the PB state of the PANI film is immersed in the mixed HCl-pyrrole solution. 1) The de-wrinkled PANI film in the PB state is immediately protonated into the protonated pernigraniline (PPN) state accompanied with the occurrence of surface wrinkling (Fig. S3b,c), once it is exposed to the mixed acid solution. This has also been effectively certified by our previous report^36^. 2) The PPN state of the wrinkling PANI film is reduced to the emeraldine salt (ES) state with the wrinkling patterns preserved^36^. 3) Simultaneously, oxidative polymerization of pyrrole into PPy occurs inside the wrinkling PANI film, just as shown in Figs 3a and S3. Namely, the following reaction happens: pyrrole + PANI (PB and/or PPN state) → PPy + PANI (ES state). Owing to the fast PB→PPN conversion combined with the subsequent rapid oxidative polymerization of pyrrole, it is not easy to trace the UV-vis absorption spectrum evolution involved in each reaction. It needs to be pointed out that once the ES state is yielded, the PANI film in the ES state has no oxidization ability to elicit the further oxidative polymerization of pyrrole. In other words, the amount of the APS-induced PB state of the PANI film determines the amount of the subsequently polymerized PPy. Their quantitative relation is supported by the evolution of the UV-vis absorption spectrum with the immersion time in the mixed pyrrole-HCl solution (Fig. S4 and the related discussion). Meanwhile, we are sure that after 2 min’s immersing, oxidative polymerization of PPy triggered by the fully oxidized state of PANI is basically terminated in the current case (Fig. S4). In order to increase the amount of the as-polymerized PPy, we can repeat the above growing cycle simply, i.e., treatment of the PDMS/PANI/PPy film with the APS solution for the regeneration of the PB state, followed by the oxidative polymerization of pyrrole in the mixed HCl-pyrrole solution. As a result, the characteristic absorbance of PPy at ∼435 nm increases with the growing cycle (Fig. 3a,b), accompanied with the surface color change from the original green to the deep black (i.e., intrinsic color of PPy). This implies that more and more PPy has been grown in the film. Interestingly, the grown amount of PPy reflected by the absorbance at 435 nm is linear to the growing cycle (Fig. 3b). As discussed above, the PANI film in the PB state acts as the template and oxidizing agent to quantitatively direct the subsequent oxidative polymerization of the embedded pyrrole. Excluding the negligible degradation of PANI from short time of treatment in the APS solution^20^, the PANI amount available to each growing cycle is constant and thus linear growth of PPy is observed (Fig. 3b). It is further supported by the result of the PDMS/PANI/PANI film (Fig. S5), where the PB state of PANI film is immersed in the mixed aniline-HCl solution to direct the additional oxidative polymerization of aniline. It is seen that exponential growth of PANI (Fig. S5) occurs because the additionally deposited PANI also acts as the oxidizing agent in the subsequent growing cycle.

Owing to the template role and the oxidizing agent of the PANI film, the oxidative polymerization of pyrrole occurs primarily inside the as-wrinkled and pyrrole/HCl-swollen PANI film, rather than on the surface of the as-wrinkled bulk film. Additionally, compared with the bulk film surface, more space from the interior swollen PANI film is available to trigger the oxidative polymerization of the internally embedded pyrrole. As a result, interpenetrated PANI-PPy networks are formed, which is very similar to our previous report of the *in-situ* self-reinforced wrinkling PPy film with the interpenetrated networks^43^. The existence of the interpenetrated PANI-PPy networks and the corresponding decisive role will be discussed subsequently in more detail.

As for the film integrity and the surface wrinkling patterns of the resulting PDMS/PANI/PPy film, they are strongly dependent on the growing cycle of PPy (i.e., the amount of the additionally grown PPy) (Figs 3c–f and S3). For example, cracks and de-wrinkling patterns occur both on the original dry PDMS_(40:1)_/PANI film (Fig. 3c) and on the APS-treated PDMS/PANI film (Fig. S3b). After the additional deposition of PPy, the cracks are gradually restrained on the PDMS/PANI/PPy film with the increase of the growing cycle (Fig. 3d–f). Especially when the growing cycle is above 4, the addition growth of PPy into PANI film leads to the formation of interpenetrated networks, lowering the volume changes of the PANI film during the air drying process to some extent. On the other hand, the formation of interpenetrated networks maybe enhances the fracture strain/stress of the PANI/PPy film. Consequently, the crack-free PANI/PPy film can be obtained (Fig. 3e,f). Meanwhile, the wrinkles on the dried film become more and more obvious with the increase of the growing cycle, indicative of the gradual increase in the wrinkle amplitude (Fig. S6) and the wrinkle stability (Fig. 3d–f). Furthermore, the wrinkle patterns on the PDMS/PANI/PPy film can withstand the treatment of the strong oxidative APS solution, although the APS-treated PANI film is converted into the PB sate (Fig. S3g). The similar enhancing effect on the film integrity and wrinkle stability also exists when PPy is replaced by PANI for the additional growth to generate the PDMS/PANI/PANI film (Fig. S7).

The above results reveal that the enhancement in the wrinkle stability and film integrity is intimately related to the additional growth of PPy and the resulting PPy-PANI interpenetrated networks. It is further supported by the control experiment, in which the *in-situ* wrinkling PDMS/PANI film was directly immersed into the mixed solution composed of oxidizing agent (e.g., APS), HCl and pyrrole (or aniline) (Supporting Information SI-S1). In this case, the existence of APS in the growing solution leads to the preferential polymerization of pyrrole (or aniline) in the solution and on the surface of the as-wrinkled PANI bulk film, while only a little of PPy (or PANI) grows inside the PANI film. Namely, in this case, the as-formed PANI/PPy (or PANI) film primarily has the laminated microstructures, rather than the above interpenetrated networks. Compared with the original *in-situ* PANI film, the wrinkle stability and the film integrity have been improved to some extent on the as-formed PANI/PPy (or PANI) film with the laminated microstructures (Supporting Information SI-S1). However, the enhancing effect of this case is by far less effective than that in the interpenetrated microstructures (Supporting Information SI-S1). It is believed that the interpenetrated networks resulting from the additional deposition of polymers inside the pre-wrinkled film would modify the stresses of the wrinkling system and thus stabilize the wrinkle patterns^43^. Note that the above mentioned pre-wrinkled patterns that will be stabilized by the additional deposition of PPy refer to the newly induced (e.g., Fig. S3b→c) or preserved (e.g., Fig. S3g→h) patterns when the APS-exposed PANI film is protonated into the PPN state by the acid mixed growing solution. Certainly, the magnitude in the modification of the stress state by the as-formed interpenetrated networks is strongly dependent on the relative content of the additional deposition of PPy related to the pre-wrinkled PANI film, just as shown in Figs 1 and 3 and Fig. S3. Recently, we have reported a self-wrinkling PPy film on the PDMS substrate with the self-stabilized two-scale wrinkling patterns^43^. It has been identified that the formation of interpenetrated networks modifies the stresses of the wrinkling PDMS/PPy system and thus stabilizes the self-wrinkling patterns. This similar stabilizing effect from the interpenetrated networks has also been observed Crosby *et al.*^17^. In their case, n-butyl acrylate (nBA) monomer was used as a swelling agent to induce surface wrinkling on a poly(n-butyl acrylate) (PnBA) substrate, followed by UV polymerization of the infiltrated nBA. They thought that the as-formed poly(n-butyl acrylate) (PnBA) was interpenetrated into the top layer of PnBA substrate with the nBA swelling-induced wrinkled patterns, leading to the formation of PnBA-locked wrinkling morphologies.

### Control of the stabilized wrinkling patterns on PANI/PPy films

**Figure 4:**
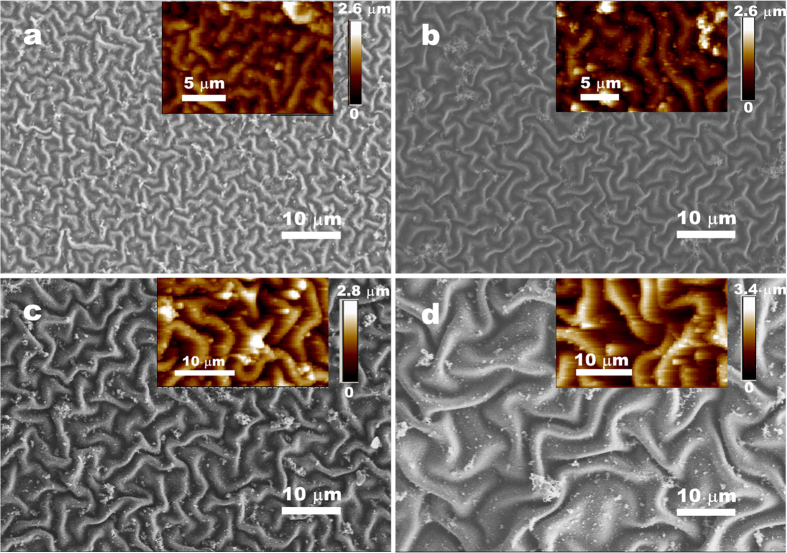
SEM images of the PDMS_(n:1)_/PANI/PPy films with n:1 = 5:1 (**a**); 8:1 (**b**); 20:1 (**c**); and 40:1 (**d**), respectively. Inset in each frame shows the corresponding AFM image.

**Figure 5:**
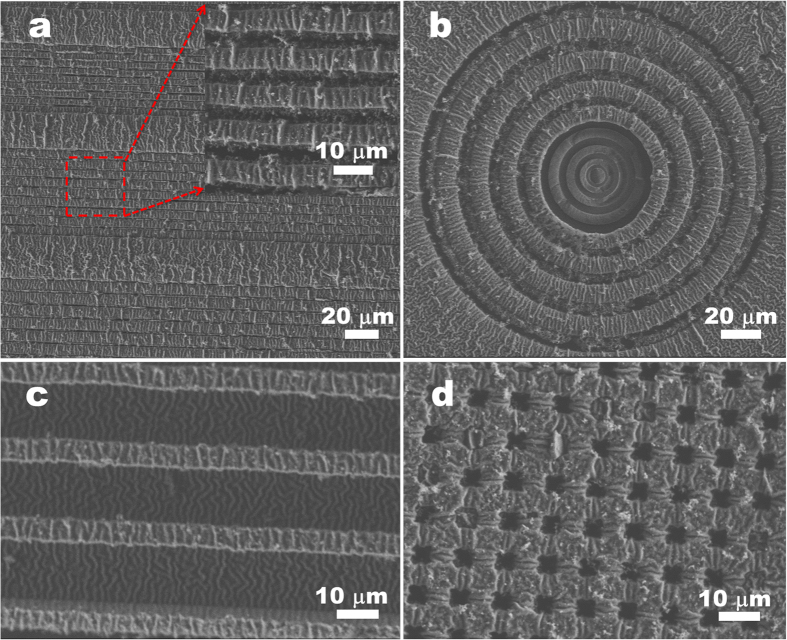
SEM images of the resulting PDMS/PANI/PPy films in the case of patterned PDMS substrates with different bas-relief microstructures applied.

Based on the template effect and the oxidizing agent of the wrinkled PANI film to direct the additional growth of PPy to form interpenetrated PPy-PANI networks (Fig. 3a,b), we can fabricate various composite films both with stable wrinkled morphologies and crack-free film integrity. During the growth of PPy in each cycle, simple and time-saving immersing processes are involved, i.e., firstly in the APS solution for 3 s and then in the mixed pyrrole-HCl solution for 2 min. In addition, only a small amount of pyrrole in the mixed solution is polymerized inside the PANI film, while most of the monomer still remains intact in the mixed solution that can be cycled. More importantly, we can finely manipulate the wrinkle patterns with controlled microstructures such as the wavelength, spatial orientation, and location (Figs 4 and 5). From the former results, we see that the additional growth of PPy has no obvious effect on the wrinkle wavelength. Thus we can adjust the original wrinkling patterns on the PDMS/PANI film to control the final patterns of the resulting PDMS/PANI/PPy film. For instance, different wavelengths of wrinkles (e.g., from 1.7 μm to 4.1 μm) are prepared by growth of PANI film on different moduli of PDMS substrates (*E*_s(n:1)_), where *E*_s(n:1)_ is tuned by the weight ratio (n:1) of the base/curing agent of PDMS. According to our previous work^36^, different wavelengths of wrinkled PANI film are easily obtained after the *in-situ* deposition of PANI on different moduli of the PDMS substrates. After growth of appropriate amount of PPy inside the wrinkled PANI films, large-area films with various wavelengths of stabilized wrinkle patterns are yielded (Figs 4 and S8). When the boundary conditions are introduced by the surface-relief PDMS substrates, crack-free PANI/PPy films with highly oriented wrinkles are also available (Fig. 5). These enhanced, ordered stress-relief wrinkles combined with the pre-scribed bas-relief microstructures make up multi-scale hierarchical patterns, which are of benefit in different applications such as lotus-leaf-like superhydrophobic surfaces.

Since the pre-wrinkled patterns that will be stabilized by the additional PPy deposition can come from the newly induced patterns when the APS-exposed PANI film is protonated into the PPN state in the acid growth solution, this versatile stabilizing strategy should be applicable to wrinkle-free PANI films. Just as expected, after a certain growing cycles, stable surface wrinkles with the good film integrity have also been generated on the initially flat PANI films, no matter the flat films are directly grown (Fig. S9) or spin-coated (Fig. S10) on the PDMS substrates (see detail in the related discussion of Fig. S10). Besides, the above strategy offers an unprecedented advantage to achieve selective growth of PPy to form hierarchical patterns, because the PANI-directed oxidative polymerization of PPy only occurs where PANI exists (Fig. S11). It is expected that other desirable polymers and materials can be introduced into the PANI film by this method to prepare well-designed copolymers and composites, which would widely extend the technological potentials of conducting polymers.

### Fabrication of a free-standing conducting film with stabilized wrinkling patterns and subsequent transfer onto a target substrate to fabricate hierarchical patterns

**Figure 6:**
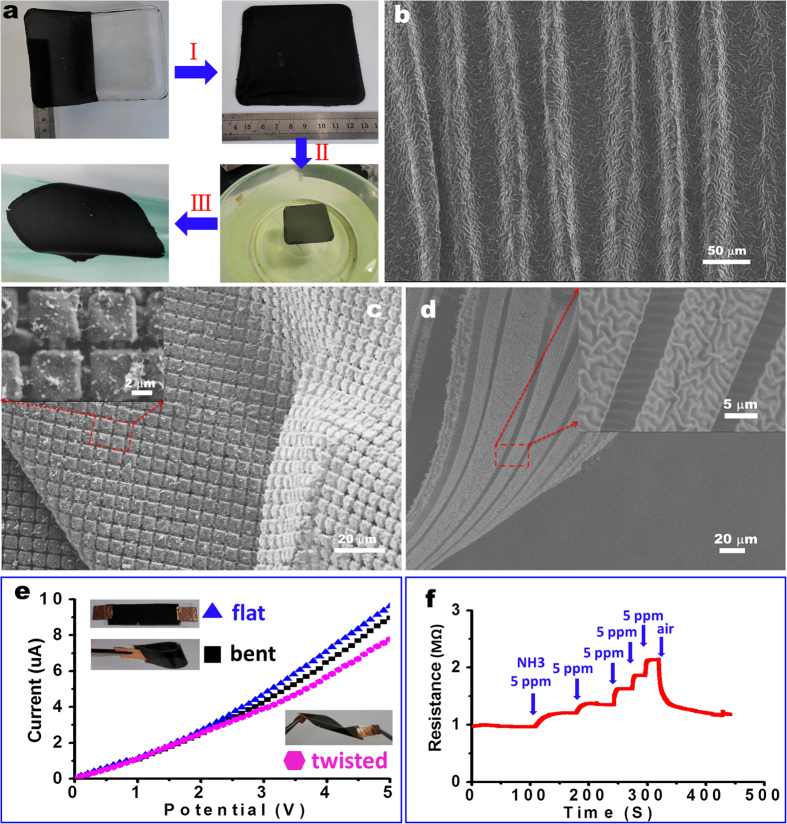
Frame a shows the process to fabricate the free-standing wrinkling PANI/PPy film: peeling the wrinkling PANI/PPy from the PDMS substrate with the help of a PVA precursor layer (I), followed by water dissolution of the PVA layer to yield a free-standing one (II) and final transfer onto target substrates (III). Frame b shows SEM image of the resulting hierarchical patterns when the free-standing wrinkling PANI/PPy film is transferred to a pre-strained PDMS substrate, followed by pre-strain release. Frames c,d show SEM images of the free-standing PANI/PPy film when the initial PANI film was *in-situ* grown on a structured PDMS substrate. Frames e, f show the conductivity and the NH_3_-response of the free-standing PANI/PPy film, respectively.

This simple strategy is conveniently applied to pattern the dry conductive film over large areas (*e.g.,* 9 × 9 cm^2^ of Fig. 6a), even in the case where curved surfaces/films are employed (Fig. S12). The resulting patterned conductive film covered on the surface of a PDMS microrod (Fig. S12) is of benefit to fabricate flexible electrode and supercapacitor^44^,^45^. Most interestingly, due to the greatly enhanced stability of wrinkling patterns on the composite film, the patterned PDMS/PANI/PPy film can be peeled from the PDMS substrate to become free-standing. Here a concentrated water-soluble poly(vinyl alcohol) (PVA) solution (e.g., 10%) is cast on the wrinkled PDMS/PANI/PPy film. After being dried in air, the PVA film combined with the wrinkled PANI/PPy film is detached from the PDMS substrate (Fig. 6aI). After dissolution of the PVA layer in water (Fig. 6aII), a self-supported crack-free PANI/PPy film with the wrinkle patterns is obtained. This free-standing film can be transferred to desired target substrates, such as ITO conducting glass, polyethylene terephthalate film (Fig. 6aIII) and interdigitated electrodes. In our observation, no obvious changes in the wrinkling patterns before and after peeling-off the film. It should be noted that the reinforcing effect from the internal growth of PPy plays a decisive role in the formation of the free-standing film with the retained wrinkling patterns (Fig. S13). Without the additional growth of PPy, the detached PANI film will crack into small pieces and no wrinkling patterns are reserved (Fig. S13a). In contrast, a large-area free-standing film with stable buckling patterns is achieved after the growth of PPy inside the PANI film (Fig. S13b). Thanks to the free-standing state of the film, both sides of the dried film can be observed (Fig. S13c,d). The wrinkling patterns maintain well on the free-standing films even when they are transferred to target substrates, which is favorable to fabricate hierarchical structures. For example, when transferring the free-standing wrinkled film to a pre-strained PDMS substrate followed by releasing the pre-strain, we obtain two-scale wrinkling structures (Fig. 6b). Due to the reinforcing effect of the subsequent PPy growth, the PANI films with other non-wrinkling patterns (e.g., *in-situ* growth of PANI film on a pre-patterned PDMS substrate), can be peeled from the substrate to become free-standing (Fig. 6c). From the sectioned side, we see the free-standing film has a small thickness (about 700 nm) (Fig. S14). In addition, free-standing films with the hybrid patterns (e.g., wrinkles combined with different bas-relief microstructures) are achieved via this simple method (Fig. 6d).

### Applications of the self-supported patterned films as flexible electrodes, gas sensors, and surface-enhanced Raman scattering substrates

The wrinkled crack-free PANI/PPy film in the free-standing state can be applied in many fields. For instance, when transferred to a piece of tape, no matter it is folded or twisted, the conductivity of the crack-free wrinkling film remain almost unchanged compared to the flat film (Fig. 6e), indicating its potential as flexible electronic devices. It also demonstrates that the free-standing wrinkling PANI/PPy film has a good sensitivity of NH_3_ gas (e.g., 5 ppm NH_3_), which can be applied as a flexible sensor (Fig. 6f). Note that when the PANI/PPy film is exposed to a high concentration of NH_3_ (e.g., 1 M NH_3_.H_2_O solution), it remains conductive still (Fig. S15). As we know, the pure PANI film will become insulated due to the NH_3_-dedoping. Thus in this case, the additionally grown PPy plays an important role in enhancing the conductivity. This kinds of hybrid films both with good electrical conductivity and high sensor responsibility have great potentials as stimulus-responsive electrical switchs^46^.

**Figure 7:**
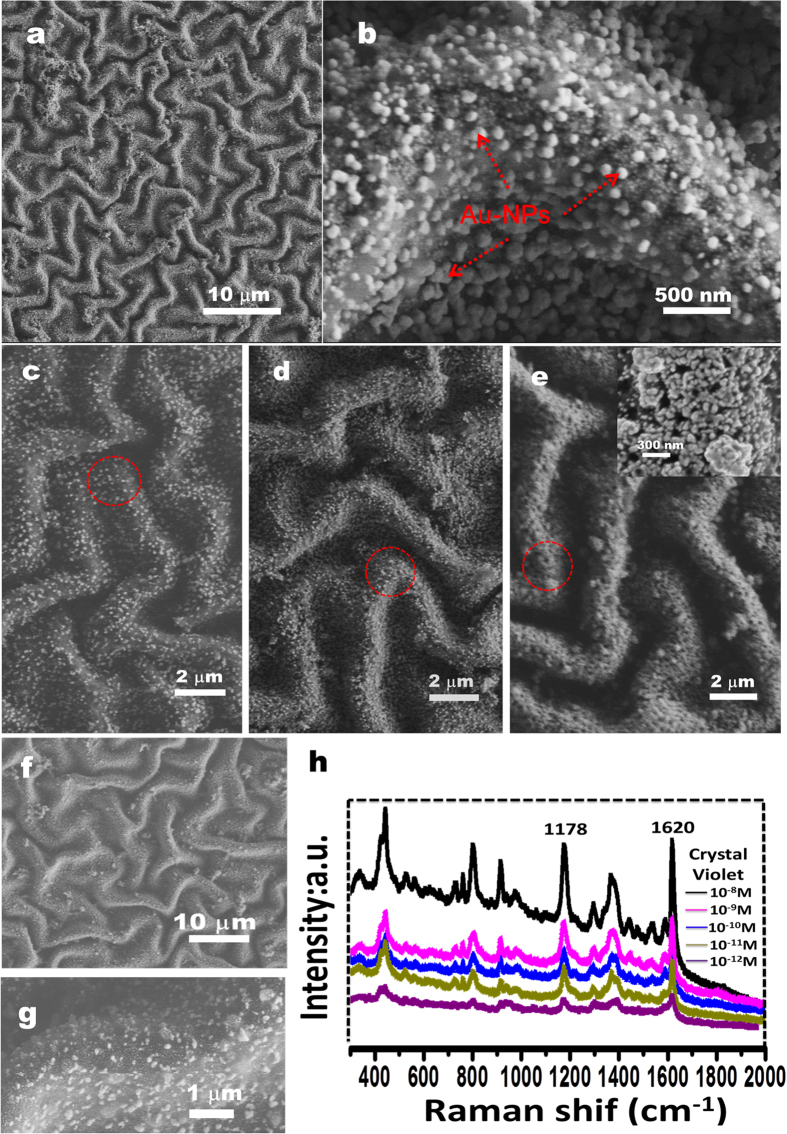
SEM images of the resulting PDMS/PANI/PPy composite film coated with Au NPs (**a–e**) and Ag NPs (**f,g**). Frame h shows the Raman measurements of different concentrations of crystal violet on the Ag NP-coated composite film.

Since the free-standing patterned film is stable, further processing the film is of benefit to expand its application range. For example, the wrinkled fluoride-treated film might be used as a flexible lotus-type super-hydrophobic coating^47^,^48^ due to the synergistic effect of microstructures and surface chemistry. The wrinkled PANI/PPy film can also be used as a template to integrate one or more components for the controlled fabrication of multi-functionalized composited films. Considering the fact that the ES state of PANI can be reduced into the state of leucoemeraldine base (LEB) by exposure to hydrazine^49^, the reducibility of the LEB state can be further employed to reduce metal salts (*e.g.*, HAuCl_4_ and AgNO_3_) for the controlled synthesis of metal nanoparticles (*e.g.*, Au nanoparticles (Au-NPs) and Ag nanoparticles (Ag-NPs))^15^,^50^. As expected, the patterned PANI/PPy film is covered with densely packed 50–120 nm sized Au-NPs (Fig. 7a,b). The sizes and numbers of the grown Au-NPs can be well tuned by the HAuCl_4_ concentration and reaction time (Fig. 7c–e, see details in the experimental section). Similarly, we also prepare the PANI/PPy/Ag-NPs film (Fig. 7f,g). These free-standing films are of great benefit as transferable SERS substrates^51^,^52^,^53^. In our case, the wrinkled conducting film integrated with Ag-NPs is applied to detect crystal violet molecule with the concentration down to 10^−12^ M (Fig. 7h). This improvement in the Raman enhancement is intimately related to the wrinkled morphology of the substrates, which is in accord with previous reports^51^,^52^. These hybrid materials are also expected to be applied as transferable biosensors^54^, photo-catalysts^55^ and photovoltaic cells^56^, and a new generation of biomaterials with outstanding comprehensive performances^30^,^31^.

## Discussion

In conclusion, we report a simple and versatile method to fabricate self-supported crack-free PANI/PPy films with stable wrinkle patterns. It is based on the template effect and the oxidizing agent of the pre-wrinkled PANI film to direct the internal growth of PPy. The resulting interpenetrated PANI-PPy networks modify the stresses of the wrinkling system, which stabilizes the surface wrinkling morphology and prohibits the crack formation. It is found that the stabilizing effect is strongly dependent on the amount of the subsequently grown PPy. Various stable wrinkling patterns on planar and curved PDMS substrates are achieved *via* the manipulation of the substrate modulus and introduction of the boundary conditions of the patterned substrate. This novel facile way is of benefit to achieve the selective growth of PPy to fabricate stable hierarchical patterns. We indicate that self-supported crack-free films with stable wrinkles are available after the substrate removal, which can be further transferred onto arbitrary target substrates to fabricate functional devices such as flexible electrodes and gas sensors, just as demonstrated here. In our case, it involves some mechanical and chemical processes in our system, further investigation of the physics underlying the formation and suitable model need to be explored. This simple versatile stabilizing strategy is highly expected to other systems for the controlled fabrication of patterned self-supported crack-free films with desirable functions and unprecedented applications. Additionally, the stable wrinkling patterns are obtained through the similar approach in the living systems, in which cell division and differentiation generate and modulate internal stresses to form stabilized hierarchical patterns^57^, such as fingerprints and the topological conformation of some fruits. Our system might serve as a model system to understand the formation of complex multi-scale micro-textures of biofilms on various substrates, in which the similar stabilizing effect may be involved^58^,^59^.

## Methods

### Preparation of Planar and Structured PDMS_(n:1)_ Substrates and the *in-situ* Wrinkling PDMS/PANI Film

The preparation of PDMS_(n:1)_ substrate and growth of *in-situ* wrinkling PANI film on PDMS_(n:1)_ substrate were carried out according to our previous reports^36^. Roughly, PDMS_(n:1)_ substrate was immersed in the mixed HCl solution composed of aniline and APS. After reaction for the designed duration, the as-prepared PDMS/PANI film was taken out, followed by water washing and air drying. Different *in-situ* wrinkling patterns of the resulting PDMS_(n:1)_/PANI film with varied wavelengths on planar and structured PDMS_(n:1)_ substrates were fabricated respectively. Unless specified otherwise, the applied PDMS_(n:1)_ substrate is from the weight ratio (n:1) of base/curing agent of 10:1, that is, PDMS_(10:1)_.

### Preparation of PDMS/PANI/PPy Film and PDMS/PANI/PANI Film by Means of the Additional Growth of PPy and PANI on the As-Prepared PDMS/PANI Film

Firstly, the above as-prepared PDMS/PANI film was immersed in the 0.1 M APS solution for 3 s, followed by water washing to remove the residual APS. Secondly, the APS-treated PDMS/PANI film was immediately immersed in the mixed solution composed of 1 M HCl and 0.1 M pyrrole (or aniline). After 2 min, the resulting PDMS/PANI/PPy film and PDMS/PANI/PANI film were taken out with a slight water washing and air drying. Subsequently, the obtained PDMS/PANI/PPy film and PDMS/PANI/PANI film were again exposed to APS solution and then the mixed solution composed of 1 M HCl and 0.1 M pyrrole (or aniline) for further growth of PPy and PANI, respectively. The growing cycle can be repeated for the designed number to adjust the deposited amount of PPy and PANI.

### Fabrication of Free-Standing Conducting PANI Film with Stabilized Patterns and Subsequent Transfer onto a Target Substrate

A 10% aqueous solution of poly(vinyl alcohol) (J&K Scientific Ltd, Mw = 20000–30000), was casted on the air dried conducting film. The PVA film coupled with the patterned film was carefully peeled from the PDMS substrate after the PVA film was dried. Subsequently, the peeled sample is floated in deionized water to dissolve the PVA layer, followed by transfer to target substrates such as a plastic sheet and a pre-strained PDMS substrate.

### Fabrication of Free-standing Wrinkling Conducting/Metal Nanoparticle Composite Films

Before casting PVA layer, the air-dried patterned conducting film was treated with hydrazine vapor, and then immersed in different concentrations of noble metal salt solutions (e.g., 0.1 M, 0.01 M and 10^−4^ M HAuCl_4_ or AgNO_3_ solution). After a designed duration (e.g. 5 s, 1 min and 10 min), the sample was taken out, followed by slight water washing and air drying. Finally, the hybrid films were peeled from the PDMS substrate with the help of PVA layer and then transferred to target substrates for the device fabrication.

### Characterization

The patterned samples were characterized by an inverted Observer A1 microscope (Zeiss, Germany) and atomic force microscope (Agilent 5500 AFM/SPM) in tapping mode. The samples without metal sputtering were observed by a scanning electron microscope (Hitachi S-4800). Evolutions of UV-vis absorption spectrum with the growing cycle of PPy were recorded on a T6 NEW CENTURY spectrophotometer. The sample conductivity was measured by Keithley 2400 sourcemeter. SERS characterization was performed when the samples were immersed in different concentrations of crystal violet (or Rhodamine B) aqueous solutions, followed by air drying. The samples were excited using a 633 nm laser line Renishaw Laser Raman Spectrometer with the power beam of 10 mW.

## Additional Information

**How to cite this article**: Xie, J. *et al.* Self-Supported Crack-Free Conducting Polymer Films with Stabilized Wrinkling Patterns and Their Applications. *Sci. Rep.* **6**, 36686; doi: 10.1038/srep36686 (2016).

**Publisher’s note:** Springer Nature remains neutral with regard to jurisdictional claims in published maps and institutional affiliations.

## Supplementary Material

Supplementary Information
