# Supplementary material for: Self-Supported Crack-Free Conducting Polymer Films with Stabilized Wrinkling Patterns and Their Applications
Source: Sci Rep. 2016 Nov 9;6:36686. doi: 10.1038/srep36686 (PMC5101525; doi:10.1038/srep36686)
Supplement: Supplementary Information [file srep36686-s1.pdf]

Supporting Information

Self-Supported Crack-Free Conducting Polymer Films with Stabilized Wrinkling Patterns and Their Applications

Jixun Xie, Xue Han, Haipeng Ji, Juanjuan Wang, Jingxin Zhao and Conghua Lu*

School of Materials Science and Engineering, Tianjin University, Tianjin 300072, P. R. China.

Corresponding Author: chlu@tju.edu.cn

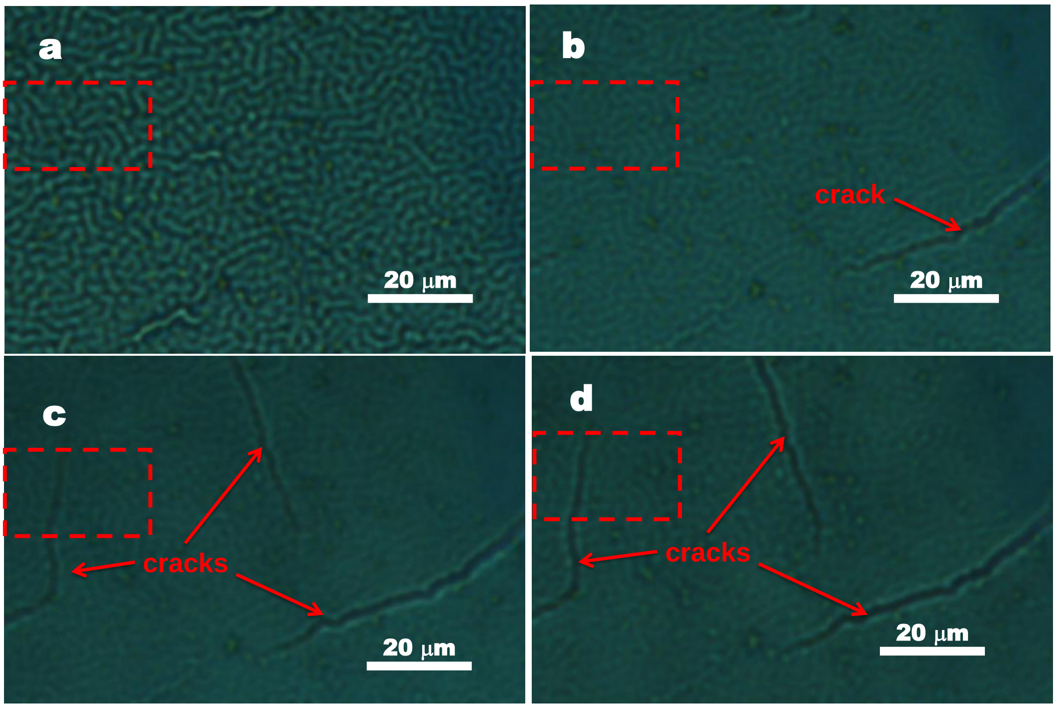

Figure S1. Morphological evolution of the as-prepared PDMS/PANI film with air drying for 1min (a); 5 min (b); 20 min (c); and 1 h (d), respectively.

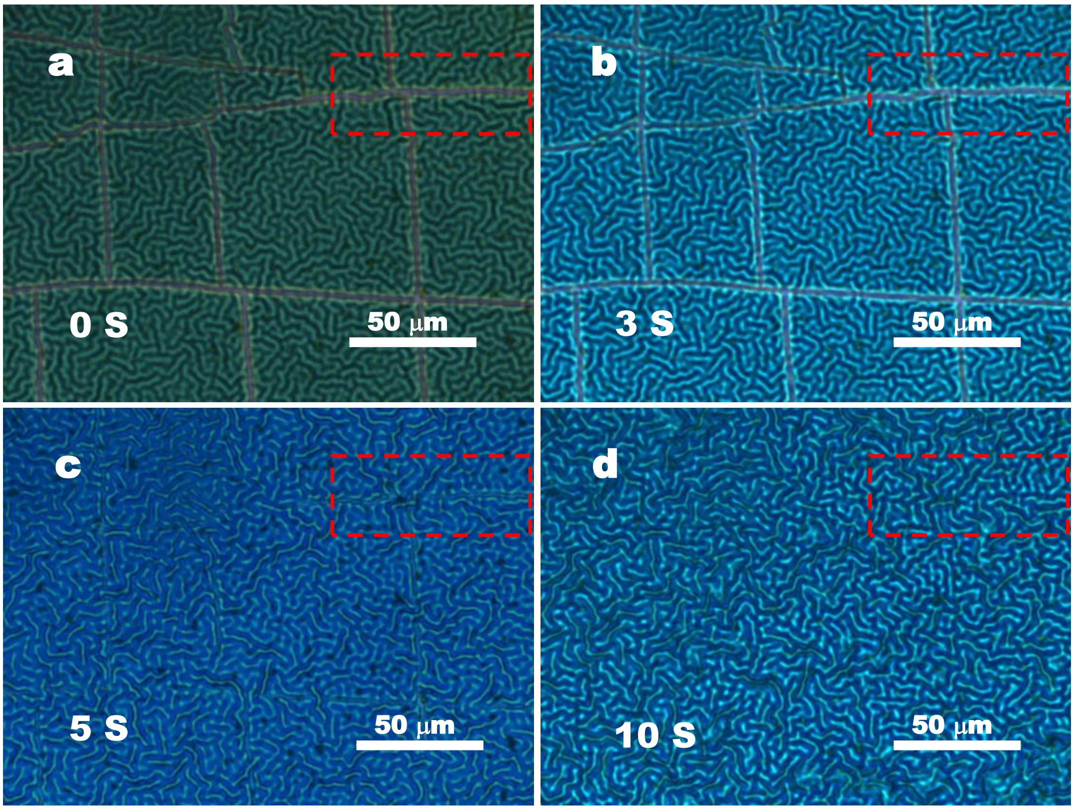

Figure S2. *In-situ* pattern evolution of the cracked PANI film (not completely dried) after dropping 1M HCl solution on its surface.

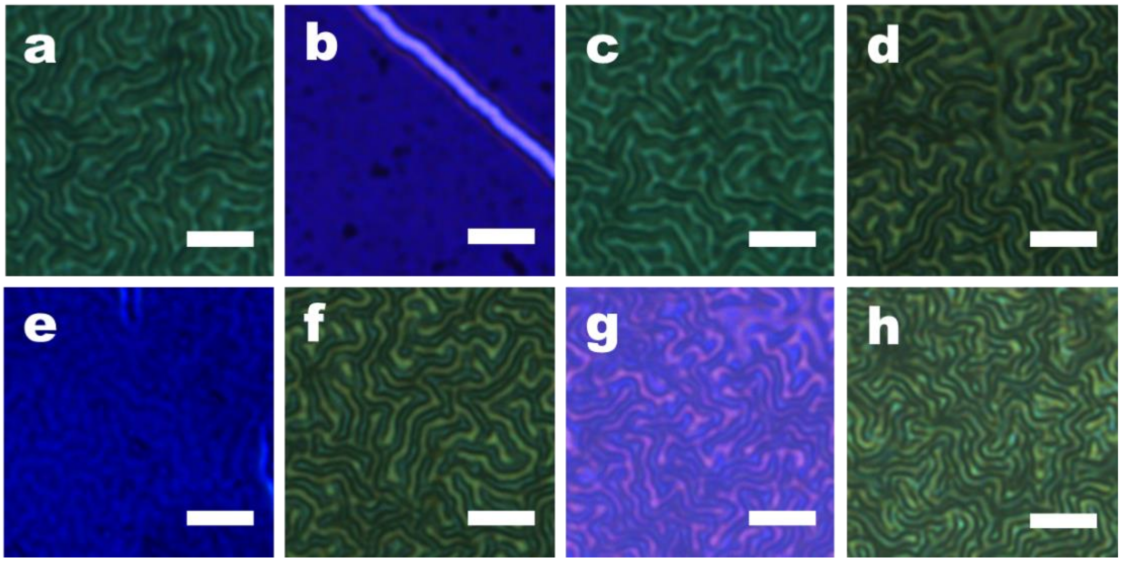

Figure S3. Optical microscope images of the PDMS/PANI/PPy film with different cycling growth of PPy. The *in-situ* wrinkling PDMS/PPy film (a) was immersed in the APS solution (b) and then in the mixed HCl-pyrrole solution for 2 s (c) and 2 min (d) for the 1st cycling growth of PPy, followed by immersing in the APS solution (e) and in the mixed HCl-pyrrole solution (f) for the 2nd cycling growth of PPy; and in the APS solution (g) and then in the mixed HCl-pyrrole solution (h) for the third cycling growth of PPy. Scale bars: 10 μm.

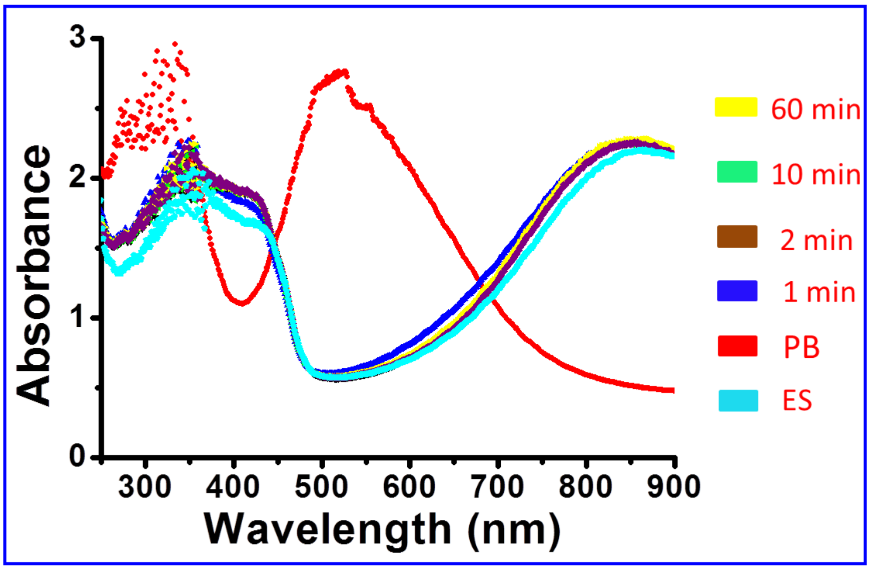

Figure S4. UV-vis absorption spectrum evolution of the APS-treated PDMS/PANI film with the reaction duration (e.g., 1 min, 2 min, 10 min and 1 h) in the mixed pyrrole-HCl solution. For comparison, the spectra of the as-prepared film before/after treatment with APS were also shown.

The *in-situ* wrinkling of PANI film grown on the PDMS substrate was in the ES state, which is supported by the blue color and the recorded typical absorption peaks at 360 nm and 820 nm[1]. When it was treated by the APS solution, the ES state was converted into the PB state, which is also supported by the purple color and the recorded typical absorption peaks at 334 nm and 550 nm[2]. Once the PB state was immersed into the HCl-pyrrole mixed solution, the PB state was protonated into the PPN state, which has been investigated in our previous paper[3]. Meanwhile, the PPN state of the wrinkling PANI film is reduced to the emeraldine salt (ES) state with the wrinkling patterns preserved[3]. Simultaneously, pyrrole is polymerized to form PPy by the full oxidization state of PANI (i.e., PB and PPN), just as shown in Figure 3a and Figure S3. Namely, the following reaction happens: pyrrole + PANI (PB/PPN state) $\rightarrow$ PPy + PANI (ES state).

It is supported by the dependence of UV-vis absorption spectrum of the resulting PDMS/PANI/PPy film with the immersion time in the mixed pyrrole-HCl solution (i.e., the polymerization duration of pyrrole and the deposited amount of PPy) (Figure S4). From Figure S4， we see that the absorbance at the absorption peak of 350-460 nm increases after immersing in the mixed HCl-pyrrole mixed solution, indicating the successful deposition of PPy on the PDMS/PANI film. However, the absorbance of the above range of absorption peaks (i.e., the deposited amount of PPy) has not increased with the immersing duration. On the one hand, it means that the oxidative polymerization of pyrrole is fast. In the current case, after 2 min’s immersing, oxidative polymerization of PPy triggered by the PB state of PANI is basically

terminated in the current case (Figure S4). On the other hand, owing to the quantitative relation between the PB state and the polymerized PPy, the constant PB content leads to the same content of PPy. As a result, we see the above dependence between the absorbance and the immersing time shown in Figure S4.

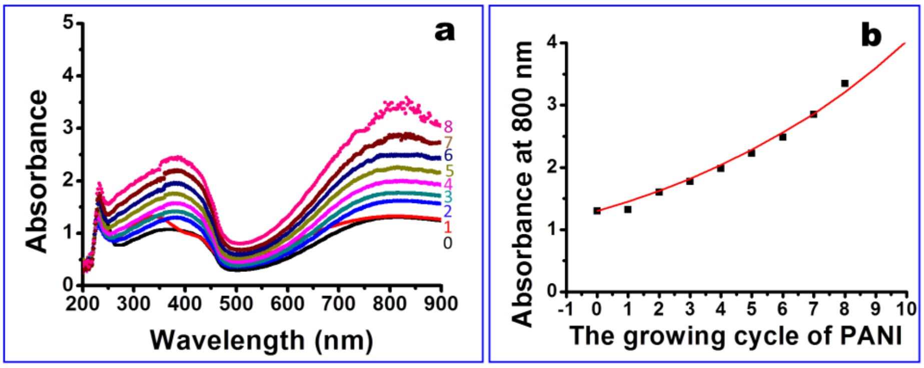

Figure S5. Evolution of UV-vis absorbance spectrum (a) of the resulting PDMS/PANI/PANI film with the cycle number of growing PANI. Frame b shows the dependence of the absorbance at 800 nm in the PDMS/PANI/PANI film on the growing cycle.

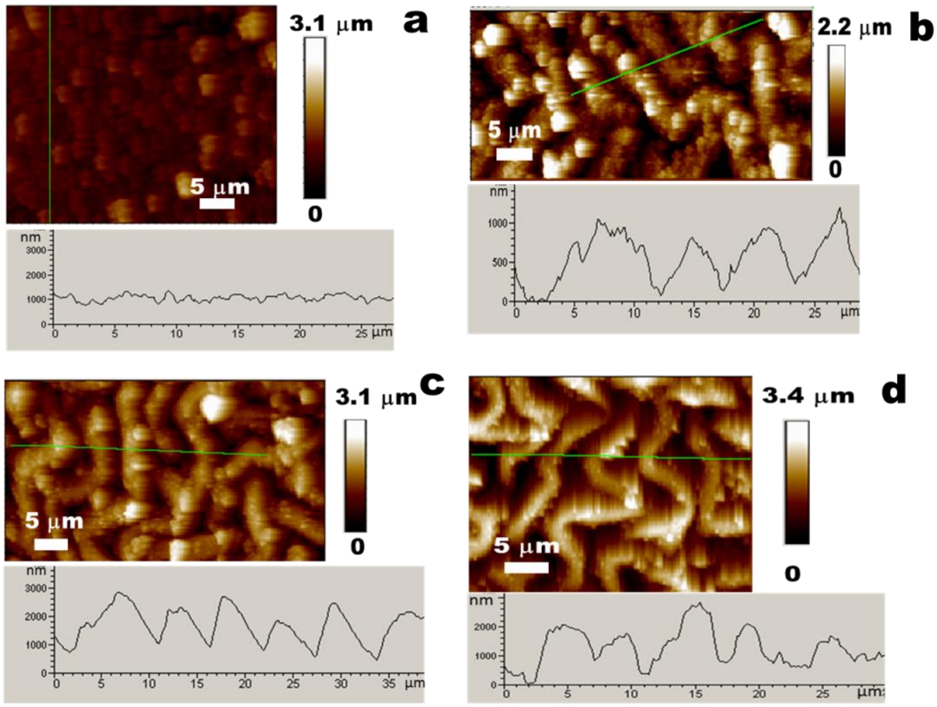

Figure S6. AFM height images and the corresponding cross-section profiles of the resulting PDMS(40:1)/PANI/PPy film from different deposition cycles. Cycle number： 0 (a)； 2 (b)； 4 (c)； 10 (d).

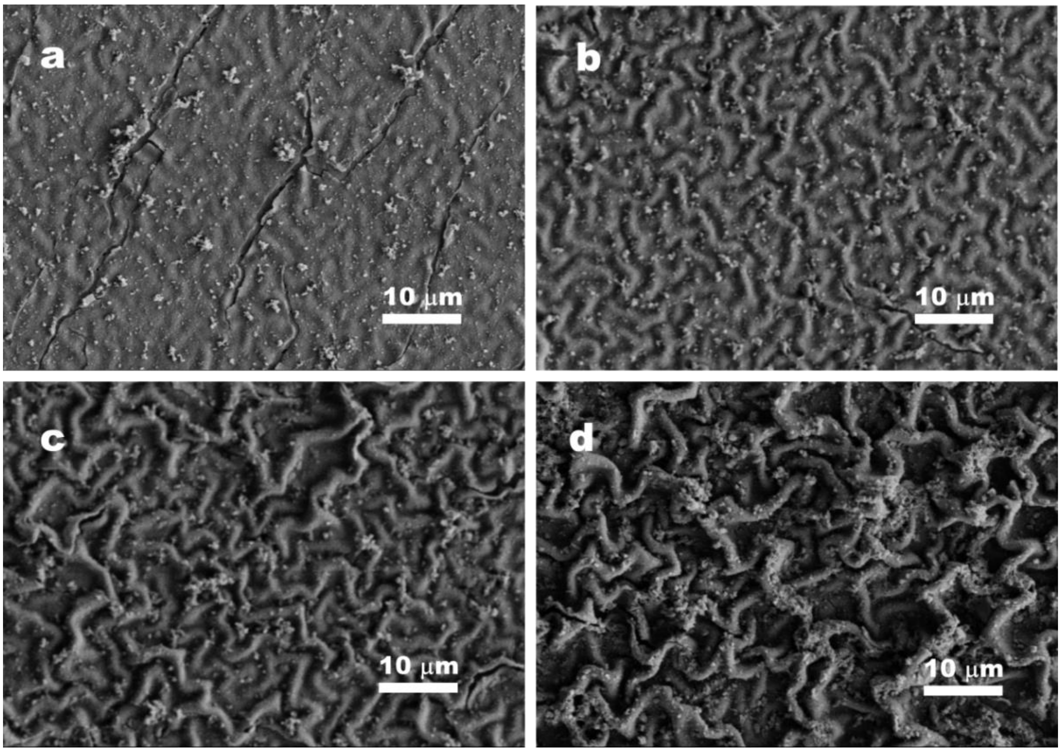

Figure S7. SEM images of the resulting PDMS/PANI/PANI film with the growing cycle number of PANI: a) 1; b) 3; c) 5 and d) 10, respectively.

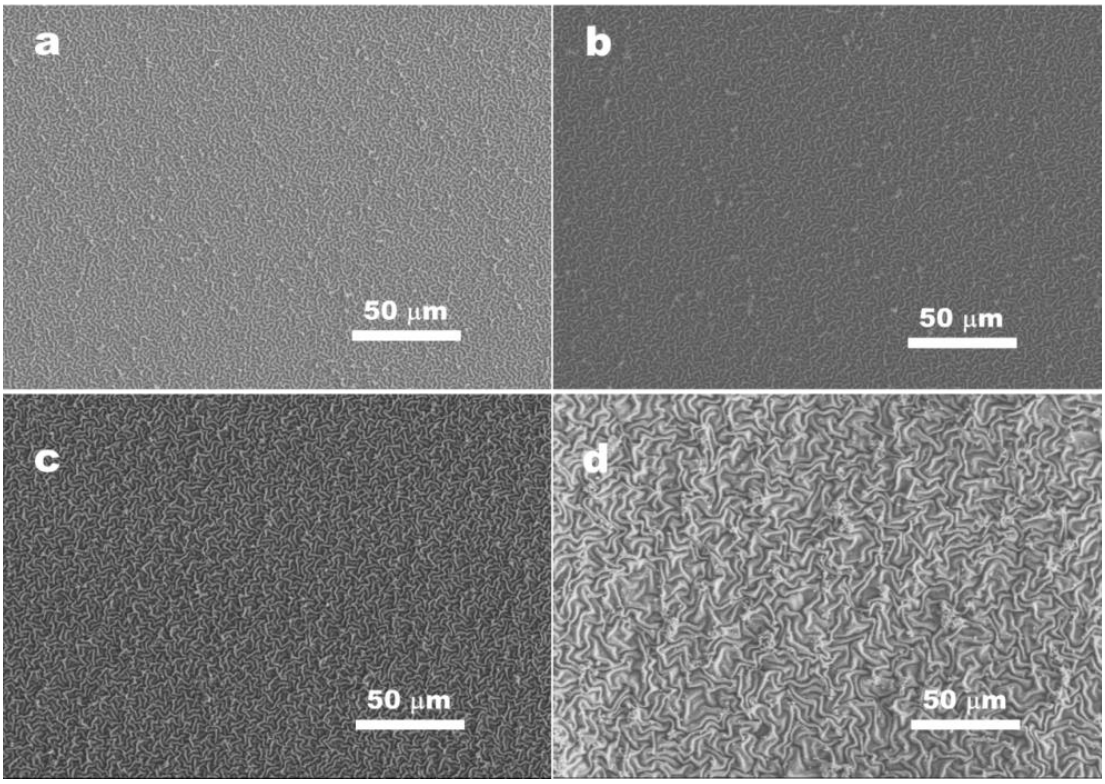

Figure S8. SEM images of the dried $PDMS_{(n:1)}$/PANI/PPy film with n:1 = 5:1 (a); 8:1 (b); 20:1 (c) ; and 40:1 (d), respectively.

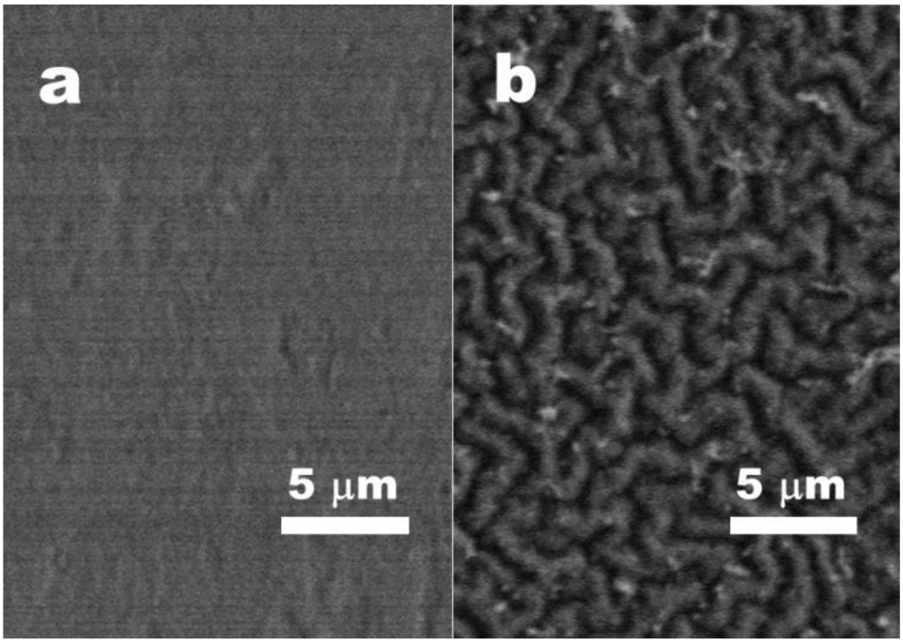

Figure S9. SEM images of the resulting PDMS/PANI film with the PANI film growth for 3 min (a), followed by growth of PPy for 8 cycles (b).

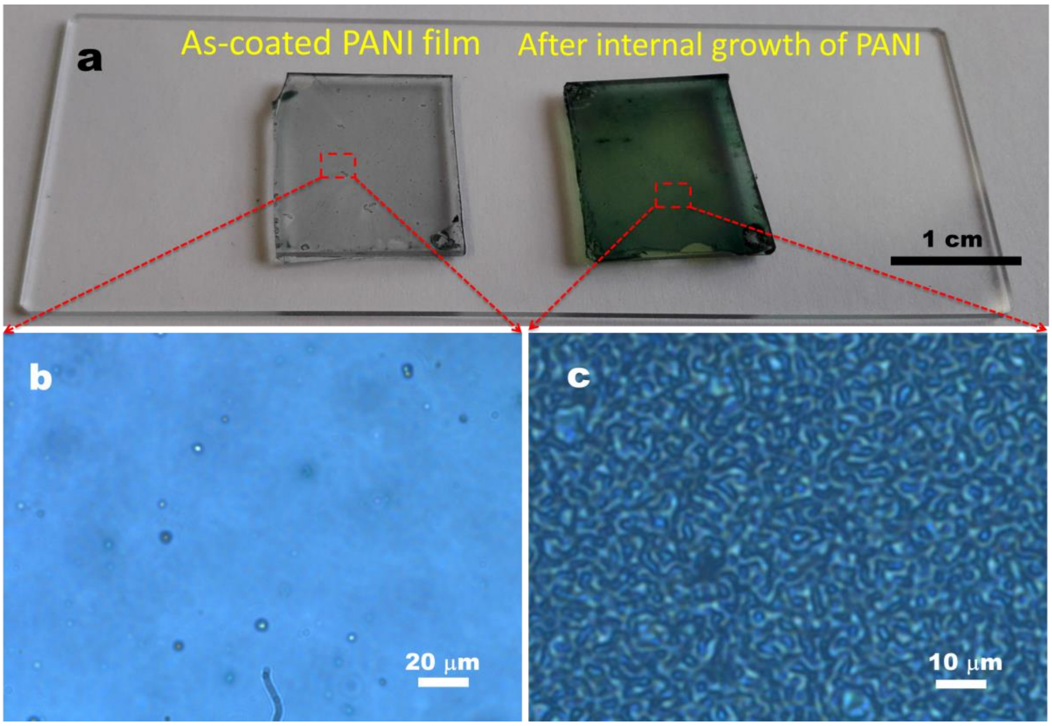

Figure S10. Digital photo and optical microscopy images of the spin-coated PANI film on the PDMS substrate (a,b), followed by internal growth of PANI (c).

It is pointed that the swelling effect of the subsequently grown PPy in the mixed acid solution plays an important role on the wrinkle formation, when the flat

PDMS/PANI film was applied to grow PPy through the above growing cycle. For instance, the PDMS/PANI film from *in-situ* polymerization of 3 min still maintains flat because the internal stress derived from swelling the thin PANI film cannot drive the *in-situ* self-wrinkling (Figure S9a)[3]. After eight growing cycles, surface wrinkles with the average wavelength of ~ 1.2 μm are formed and stabilized on the air-dried PDMS/PANI/PPy film (Figure S9b). During the growing cycles, different swelling between the PANI/PPy film and the inert PDMS substrate induces the required compressive stress and leads to surface wrinkling[3]. The subsequently grown PPy film interpenetrates into the wrinkling bulk film, resulting in an "interpenetrated polymer network" and thus stabilizing the wrinkling structures. Besides the *in-situ* grown PANI film, the spin-coated PANI film on the PDMS substrate can also be wrinkled and stabilized through this method (Figure S10), *i.e.*, alternately immersing in an APS solution and in a mixed aniline-HCl solution. Similarly, the as-coated flat PANI film on PDMS became wrinkled after several growing cycles of PANI by taking the flat PANI film as both the oxidizing agent and substrate.

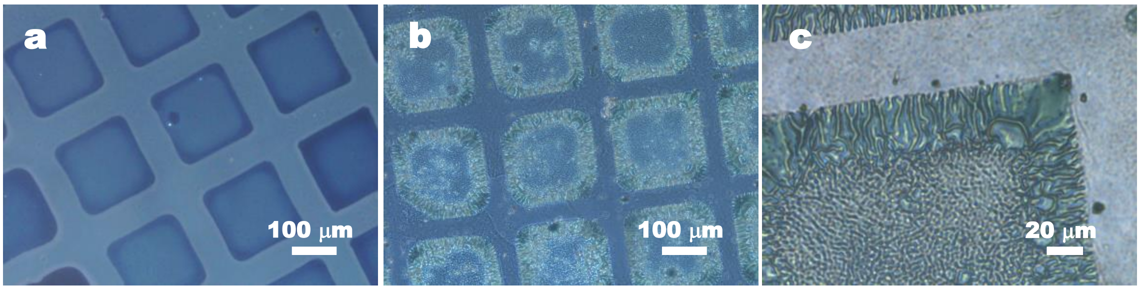

Figure S11. Optical microscopy images of the spin-coated PANI film on oxygen plasma-selectively-exposed PDMS substrate (a), followed by growth of PANI (b,c).

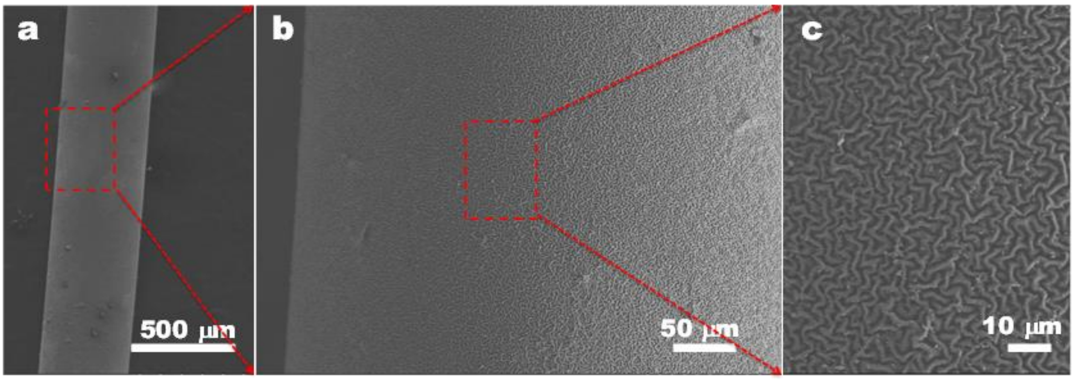

Figure S12. SEM images of the resulting PDMS/PANI/PPy film when a PDMS micro-rod was used as the compliant non-planar substrate.

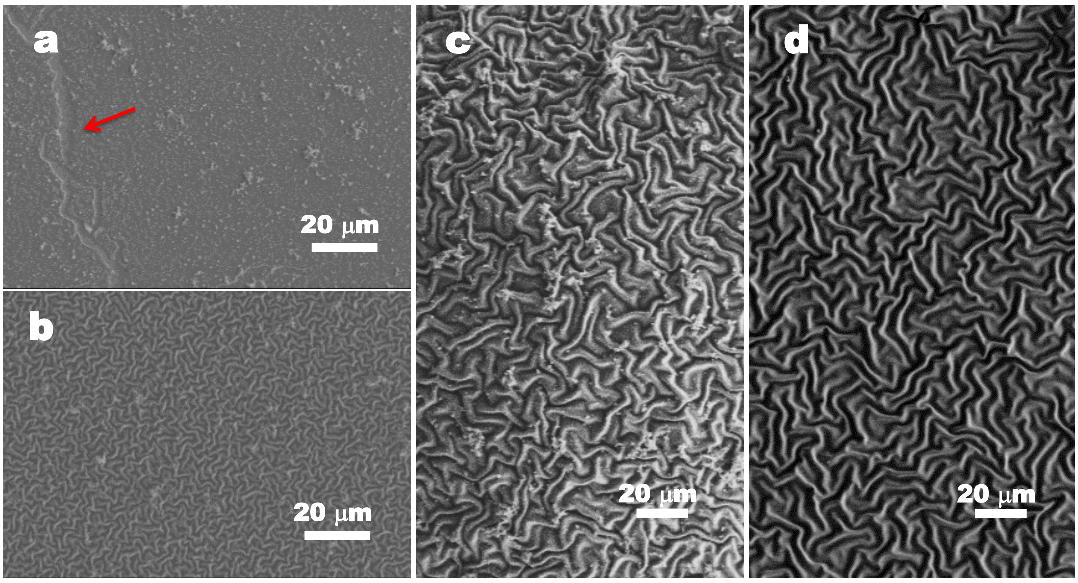

Figure S13. SEM images of the pure PANI film (a) and the PANI/PPy film (b-d) when they were transferred from the PDMS substrate onto a glass substrate. Frame c and d show the SEM images of the PANI and PPy sides of the free-standing film, respectively.

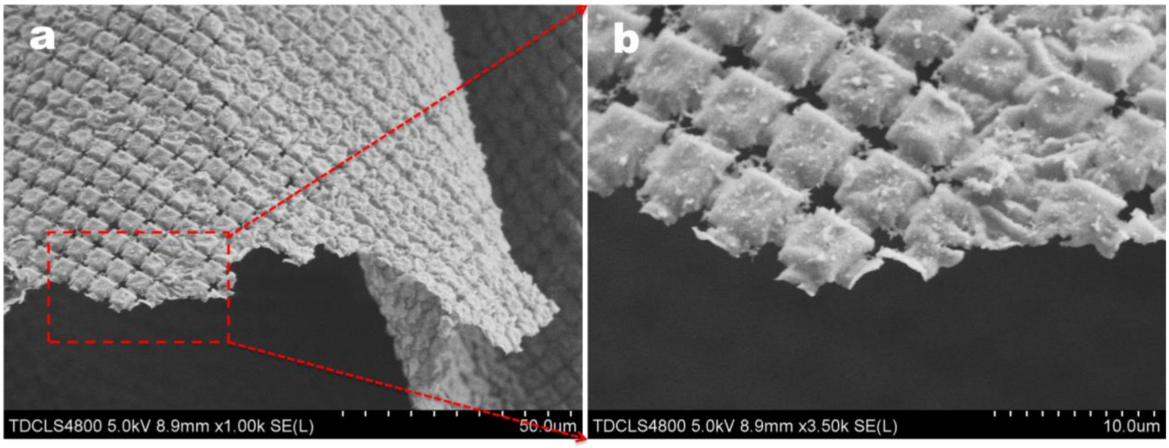

Figure S14. SEM images of the free-standing patterned PDMS/PANI/PPy film when a patterned PDMS substrate was used.

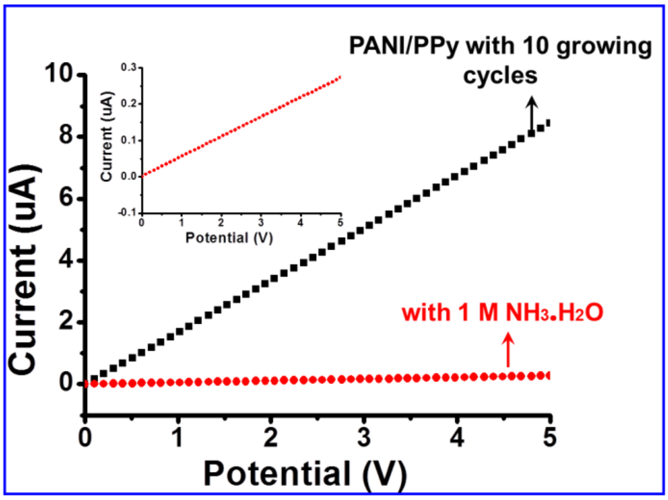

Figure S15. Plot of the current as a function of potential after the PDMS/PANI and PDMS/PANI/PPy films were treated with 1M $NH_3.H_2O$, respectively.

Supporting Information S1

This simple method, using the as-prepared PANI film as the oxidizing agent and template for the additional deposition, holds the great advantage for fabricating stable wrinkles. In a control experiment, where the wrinkled PDMS/PANI film with the PANI film in the ES state was immersed into the mixed HCl-pyrrole solution composed of an oxidizing agent (e.g., APS) for the additional deposition of PPy, we have not observed obvious wrinkles on the resulting dried PDMS/PANI/PPy film (Figure SI-1a,b). This is in sharp contrast with the case where the as-prepared PANI film is as the oxidizing agent for the growth of PPy film shown in Figure 1f. This difference might be attributed to the different growth mechanism of PPy in the above two cases. With the strong oxidizing agent of APS added in the mixed pyrrole/HCl solution, polymerization of pyrrole happened mostly in the mixed solution and on the surface of the as-formed PANI film, while only a little of PPy grew inside the PANI film. Certainly, the grown PPy in the solution has no effect on the stability of the original wrinkling patterns of PANI film. In addition, PPy aggregates formed in the solution will attach on the PANI film (Figure SI-1b), which also covers up the underlying patterns of PANI film. As a result, only fuzzy wrinkles can be discerned (Figure SI-1b). When the as-prepared PANI film acted as the oxidizing agent, most of PPy grew inside the bulk film, because polymerization of PPy needs the oxidizing agent. This comparison results indicate that the wrinkled PANI film as the oxidizing agent to achieve the internal growth of PPy is the key to stability-enhancing patterns.

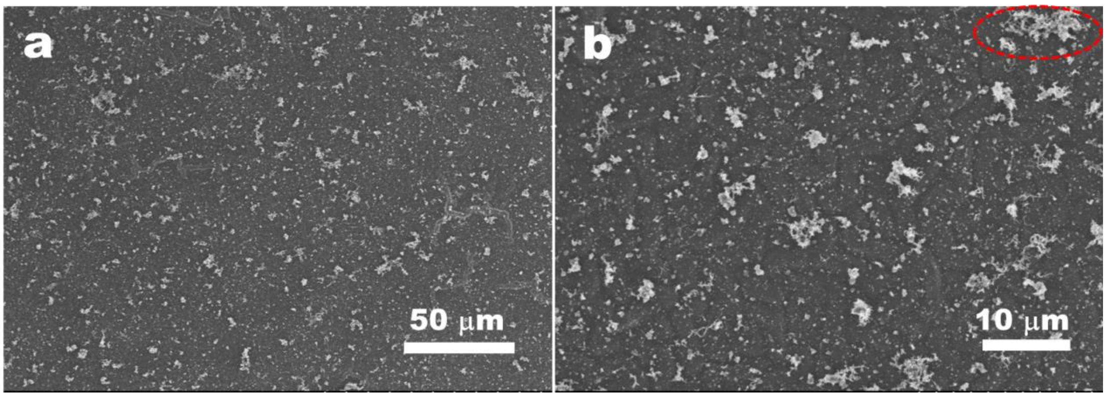

Figure SI-1 SEM images of the resulting PDMS/PANI/PPy film when the additional growth of PPy was performed in the mixed HCl-pyrrole solution composed of the oxidizing agent of APS.

Another control experiment was carried out by putting the wrinkled PDMS/PANI film into the mixed HCl solution composed of aniline and APS for the additional growth of PANI. After re-deposition of PANI on the original PANI film, partial wrinkles can be observed on the dried PDMS/PANI/PANI film (Figure SI-2). More importantly, many cracks (Figure SI-2) still existed in the dried PDMS/PANI/PANI film. In contrast, when the as-prepared PANI film was used as the oxidizing agent and substrate to internally grow PANI, obvious wrinkling patterns and few cracks occurred (Figure S7).

The above control experiments strongly indicate that the additional internal growth of polymers owing to the oxidizing effect and the templating effect of the wrinkled PANI film is of great benefit to obtain an intact conducting film with stable wrinkle patterns.

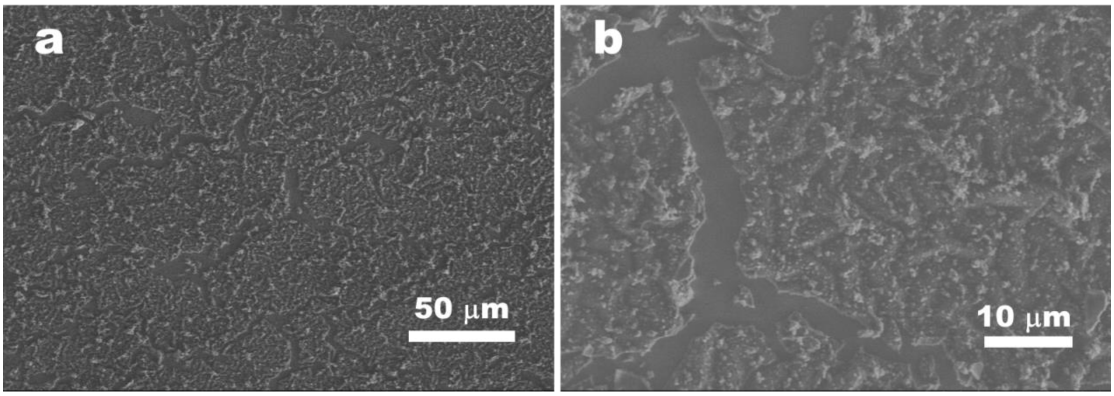

Figure SI-2 SEM images of the resulting PDMS/PANI/PANI film when the additional growth of PANI was performed in the mixed HCl-aniline solution composed of the oxidizing agent of APS.

Reference

1. Stejskal, J., Kratochvil, P. & Radhakrishnan, N. Polyaniline dispersions 2. UV-Vis absorption spectra. *Synth. Met.* **61,** 225-231 (1993).

2. Kolla, H. S., Surwade, S. P., Zhang, X., MacDiarmid, A. G. & Manohar, S. K. Absolute molecular weight of polyaniline. *J. Am. Chem. Soc.* **127,** 16770-16771 (2005).

3. Xie, J., Han, X., Zong, C., Ji, H. & Lu, C. Large-area patterning of polyaniline film based on in situ self-wrinkling and its reversible doping/dedoping tunability. *Macromolecules* **48,** 663-671 (2015).
